# Recent Advancements in Neuroimaging‐Based Alzheimer's Disease Prediction Using Deep Learning Approaches in e‐Health: A Systematic Review

**DOI:** 10.1002/hsr2.70802

**Published:** 2025-05-05

**Authors:** Zia‐Ur‐Rehman, Mohd Khalid Awang, Ghulam Ali, Muhammad Faheem

**Affiliations:** ^1^ Faculty of Informatics and Computing (FIK) Universiti Sultan Zainal Abidin (UniSZA) Besut Terengganu Malaysia; ^2^ Department of Computer Science University of Okara Okara Pakistan; ^3^ School of Technology and Innovations University of Vaasa Vaasa Finland; ^4^ VTT Technical Research Centre of Finland Espoo Finland

**Keywords:** alzheimer's disease, deep learning, deep belief network, generative adversarial network, Internet of things, magnetic resonance imaging

## Abstract

**Purpose:**

Alzheimer's disease (AD) is a severe neurological disease that significantly impairs brain function. Timely identification of AD is essential for appropriate treatment and care. This comprehensive review intends to examine current developments in deep learning (DL) approaches with neuroimaging for AD diagnosis, where popular imaging types, reviews well‐known online accessible data sets, and describes different algorithms used in DL for the correct initial evaluation of AD are presented.

**Significance:**

Conventional diagnostic techniques, including medical evaluations and cognitive assessments, usually not identify the initial stages of Alzheimer's. Neuroimaging methods, when integrated with DL techniques, have demonstrated considerable potential in enhancing the diagnosis and categorization of AD. DL models have received significant interest due to their capability to identify AD in its early phases automatically, which reduces the mortality rate and treatment cost of AD.

**Method:**

An extensive literature search was performed in leading scientific databases, concentrating on papers published from 2021 to 2025. Research leveraging DL models on different neuroimaging techniques such as magnetic resonance imaging (MRI), positron emission tomography, and functional magnetic resonance imaging (fMRI), and so forth. The review complies with Preferred Reporting Items for Systematic Reviews and Meta‐Analyses (PRISMA) guidelines.

**Results:**

Current developments show that CNN‐based techniques, especially those utilizing hybrid and transfer learning frameworks, outperform conventional DL methods. Research employing the combination of multimodal neuroimaging data has demonstrated enhanced diagnostic precision. Still, challenges such as method interpretability, data heterogeneity, and limited data exist as significant issues.

**Conclusion:**

DL has considerably improved the accuracy and reliability of AD diagnosis with neuroimaging. Regardless of issues with data accessibility and adaptability, current studies into the interpretability of models and multimodal fusion provide potential for clinical application. Further research should concentrate on standardized data sets, rigorous validation architectures, and understandable AI methodologies to enhance the effectiveness of DL methods in AD prediction.

## 1 Introduction

**Figure 1:**
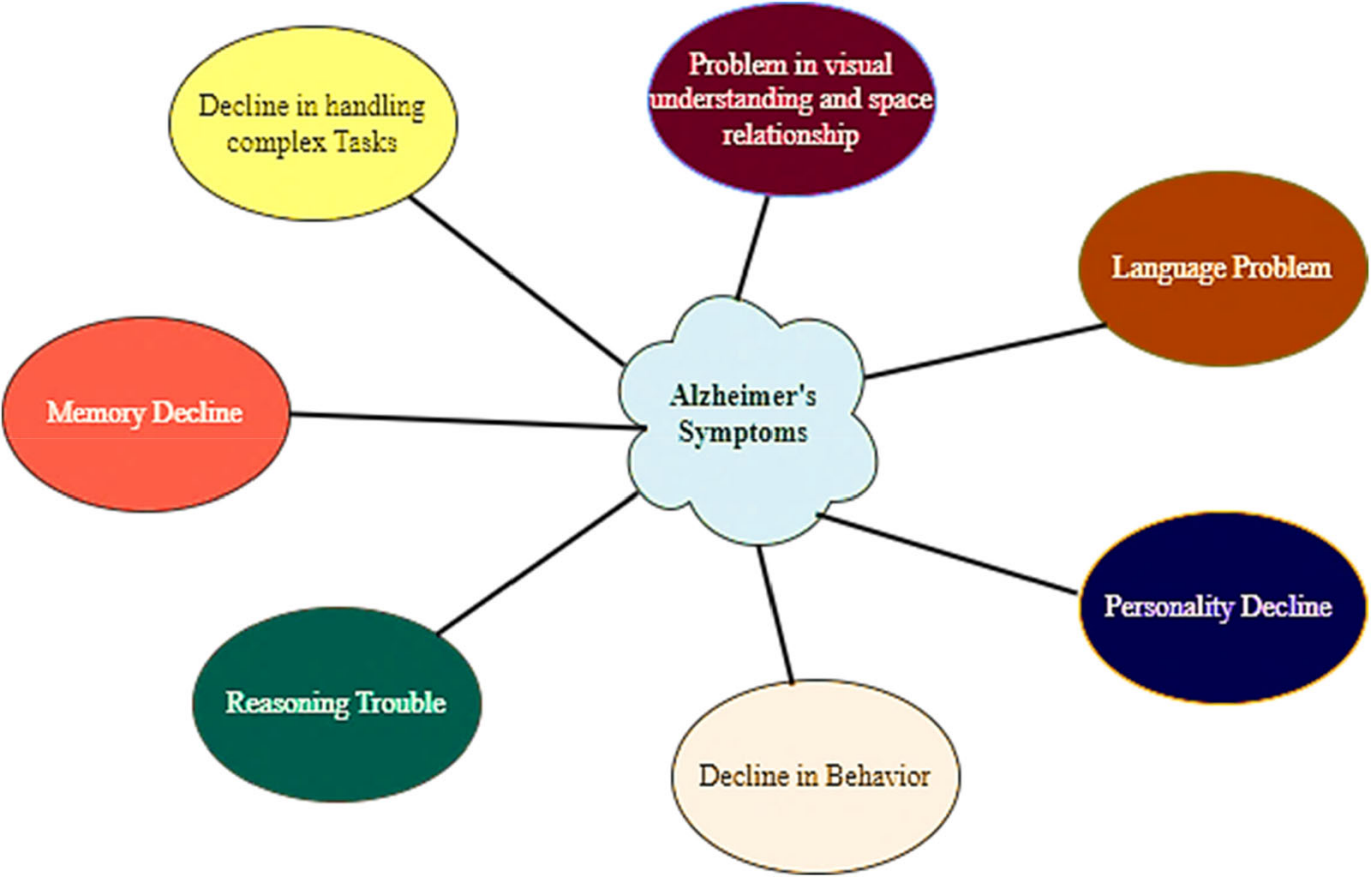
Different symptoms of AD.

The human brain is a highly complex and vital body element, making its disorders significant from a medical perspective. Disorders in the brain lead to different complications, and one of them is Alzheimer's. Alzheimer's disease (AD) is the most common type of dementia. AD is a degenerative neurological disease that deteriorates over time and is irreversible. It is defined by a decline in memory, deterioration in cognitive abilities, and challenges in everyday tasks [1, 2]. Seven stages comprise AD, from normal to severe AD [3]. Figure 1 shows the different signs of AD. Initial detection of the disease facilitates appropriate patient treatment, therefore reducing the detrimental impact of AD and slowing the progression toward dementia [4]. The deaths and cost are also critical aspects of AD as in 2010; dementia was a disease that affected 35.6 million individuals who were 60 years old or older worldwide. Estimates suggest that this number will double each decade, exceeding 115 million in 2050 [5]. Dementia has emerged as the second most common reason for mortality in Australia, resulting in notable financial impacts [6]. Although several treatment approaches have been investigated, their effectiveness has been restricted, highlighting the significance of timely and precise identification of suitable treatment options [7]. Therefore, the early detection of AD is crucial. Brain imaging, especially structural magnetic resonance imaging (SMRI), detects AD by visualizing the shrinkage of brain structure. The sMRI offers detailed benefits and availability for examining and monitoring its probable cause [8]. Neuroimaging biomarkers are crucial in providing precise analysis and rapid AD detection. Many studies have focused on machine‐learning methods for identifying AD. Nevertheless, conventional machine learning methods have encountered problems when handling the complexities of detecting AD. Recently, there have been notable improvements in deep learning (DL) methods driven by the increased computational power of graphics processing units [9]. This article thoroughly examines the recent state of the art of AD identification utilizing DL approaches. Our objective is to investigate the utilization of DL in supervised and unsupervised techniques to get a broader understanding of AD.

## 2 Research Methodology

**Figure 2:**
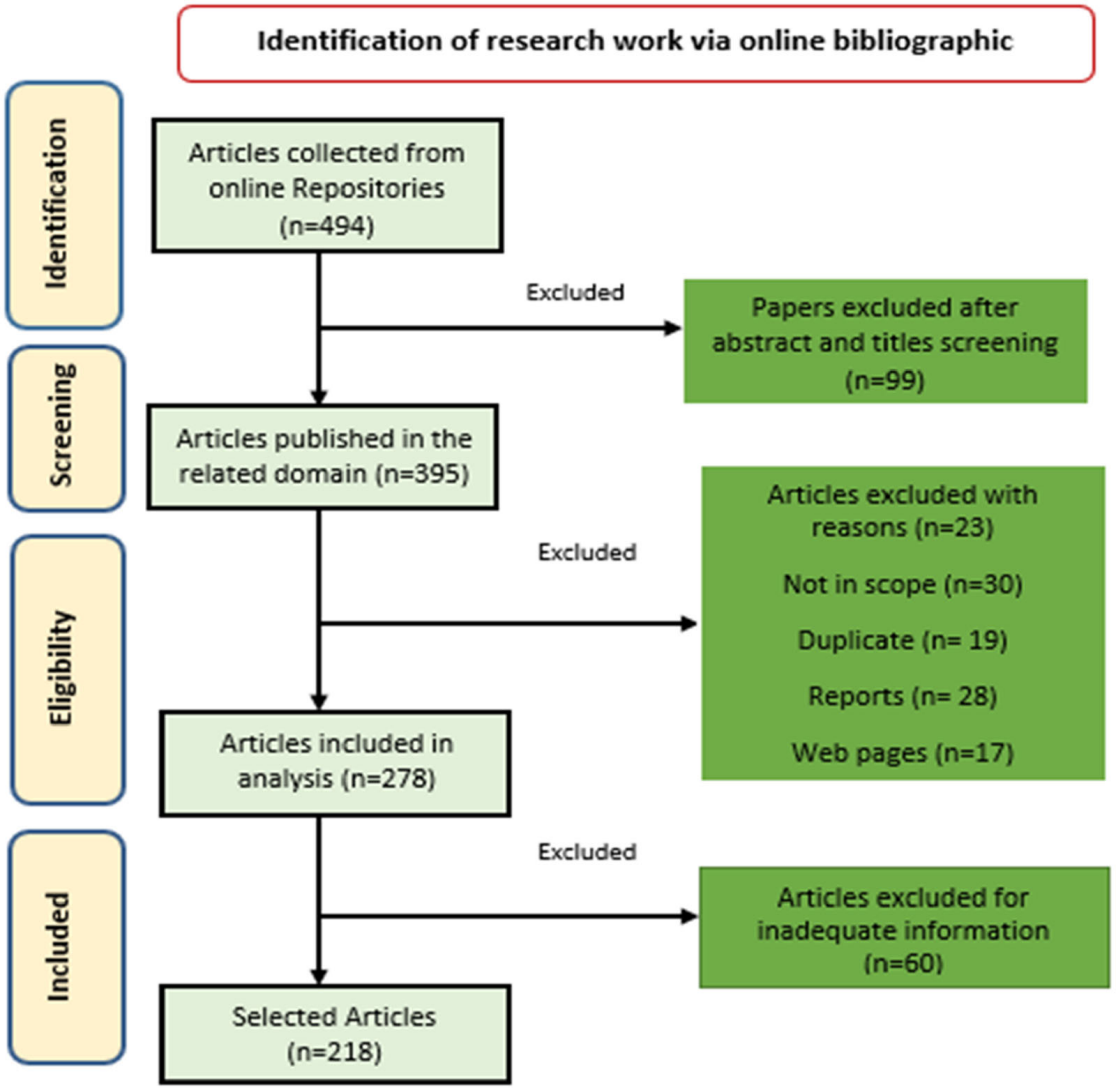
PRISMA flow diagram of the paper selection process.

This study's methodology follows the requirements specified in Figure 2 of the PRISMA, guaranteeing an organized and perfect technique for combining data. PRISMA, a well‐established practical framework for complete reviews, provided procedures for collecting critical materials for this review. Moreover, it delineates the criteria for including and excluding data in PRISMA. The inclusion and exclusion criteria give a thorough overview of the factors used to decide whether to include an item in the review or exclude it from the evaluation. At the onset of the research, we gathered articles from online bibliographic databases and chose 494 articles for the review. We conducted a comprehensive study and selected 218 publications for review. Among these, 60 papers presented distinct concepts, definitions, and explanations of models and theories, while the remaining 158 papers focused mainly on the uses, problems, and current achievements in the field.

### 2.1 Data Bases

The research examines the acknowledgment of efforts to detect AD in several scientific databases, including IEEE Xplore, Science Direct, Elsevier, Springer Link, Wiley Inter Science, Google Scholar, PubMed Central, ACM, and other electronic databases.

### 2.2 Search Terms

The analysis focuses on papers that include keywords such as “Deep learning,” “Deep learning in Alzheimer's,” “Alzheimer's diagnosis using deep learning,” “Alzheimer's diagnosis using unsupervised deep learning,” “Alzheimer's diagnosis using supervised deep learning.” In this review article, we examine papers from 2021 to 2025. We search research papers regarding AD detection using DL up to 25 pages of Google Scholar.

### 2.3 Inclusion Criteria Search

The following inclusion criteria applied when choosing articles for this review:

- Articles published from 2021 to 2025.
- Papers are presented in English.
- AD detection and DL.
- Papers published in academic journals.
- DL in medical imaging.
- Not restricted to a definite area.

### 2.4 Exclusion Criteria Search

The following exclusion criteria were used when choosing articles for this review:

- Thesis, Communication letters, white papers, editorials, and reports.
- Duplicate and non‐English articles.
- Not related to the topic of the review.
- Traditional and statistical methods.
- Articles that are absent of data.

### 2.5 Previous Research Gaps

The earlier review articles on the detection of AD using DL techniques have added contributions by presenting the most advanced methods, emphasizing the potential of these methods in early detection, and tracking the development of the disease. Still, there are numerous flaws in the existing literature work. Primarily, although numerous studies [10, 11, 12] concentrate on developing methods for detecting AD, there is an absence of research into efficient and accurate initial detection of AD is necessary to provide immediate alternatives for treatment. Currently, there is a lack of treatment options for AD, demanding a resolution for the considerable volumes of imaging data needed to handle a large patient population. An important aspect is the analysis of longitudinal data. AD is a progressive degeneration disorder, and monitoring the evolving changes is crucial for understanding its development. Several ADNI‐trained DL algorithms are unsuccessful in generalizing among imaging types, clinical, and demographic contexts. Automatic techniques are needed to handle the significant amount of patients' clinical imaging input. Raw images are highly dimensional; however, existing feature extraction methods can be either heuristic‐based or computationally costly. DL algorithms often ignore non‐imaging data, which are critically important for obtaining an accurate diagnosis of AD. Most of the research emphasizes a single type of imaging instead of combining other modalities to enhance predictability. Different research employs disparate performance criteria lacking a uniform benchmark for assessment. Limited investigation of ethical problems and practical issues when utilizing frameworks for clinical processes, regardless of technological development. Addressing these gaps is crucial for advancing the field and facilitating the translation of DL‐based AD detection methods into clinical practice.

### 2.6 Contributions

The review article has the following contributions:

- Concise overview and medical observations of the significant neuroimaging techniques used for diagnosing AD from 2021 to 2025.
- Examination of the accessible data sets used for the identification of AD.
- Analysis of several current research on supervised and unsupervised DL methods for AD detection.
- 2D & 3D imaging explored with DL techniques to identify AD and its pros and cons.

This review further discusses popular neuroimaging modalities and their comparative analysis, different data sets used in AD, AD with DL techniques (supervised and unsupervised learning), 3D imaging, discussion and challenges in AD, and conclusion and future extensions in this area.

## 3 Imaging Modalities

Previous research has established multiple imaging procedures that aid in AD identification. Nevertheless, specialists' most widespread and widely recognized techniques are positron emission tomography (PET), MRI, functional MRI (fMRI), diffusion tensor imaging (DTI), and single photon emission computed tomography (SPECT). This review provides a concise explanation of this imaging.

### 3.1 MRI

**Figure 3:**
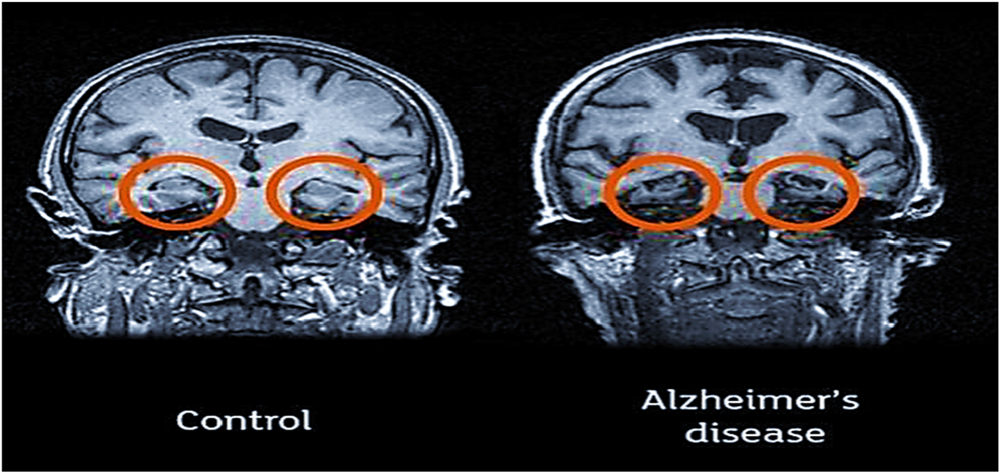
The brain sMRI scan left to right (normal and Alzheimer's affected) [16].

MRI represents a notable advancement in clinical imaging. MRI offers high spatial resolution and the ability to visualize superficial tissues with remarkable clarity [13]. In an MRI scan for diagnosing AD, very detailed images of the brain are captured. These images enable clinicians to examine characteristic features like atrophy and the shrinking of specific brain regions, frequently observed in individuals with AD [14]. In addition, structural MRI assists in promptly detecting AD by identifying minor structural alterations [15]. Figure 3 represents the sMRI.

### 3.2 fMRI

**Figure 4:**
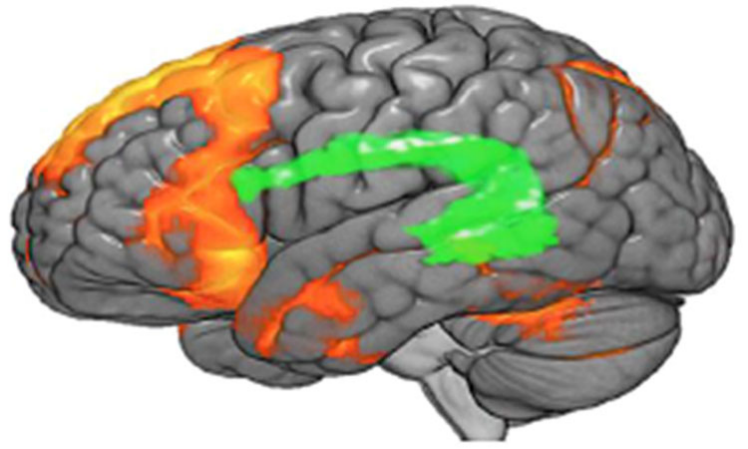
The fMRI scan of a human brain [18].

fMRI is a neurological modality that evaluates and explores neural function by identifying variations in the flow of blood. fMRI, patients engage in mental activities or remain at rest as their brain function is monitored. In people with AD, fMRI reveals abnormalities in the brain regions associated with cognition and memory [17]. It particularly emphasizes reduced activity in the hippocampus and other areas associated with it [18]. These shifts may serve as early indicators of the disease, aiding in its timely identification. In addition, fMRI is useful for monitoring the progression of diseases [19]. Due to its noninvasive nature and ability to visualize functional brain activity, fMRI is an essential tool for advancing our knowledge and treatment of AD [20]. Figure 4 shows the fMRI.

### 3.3 DTI

**Figure 5:**
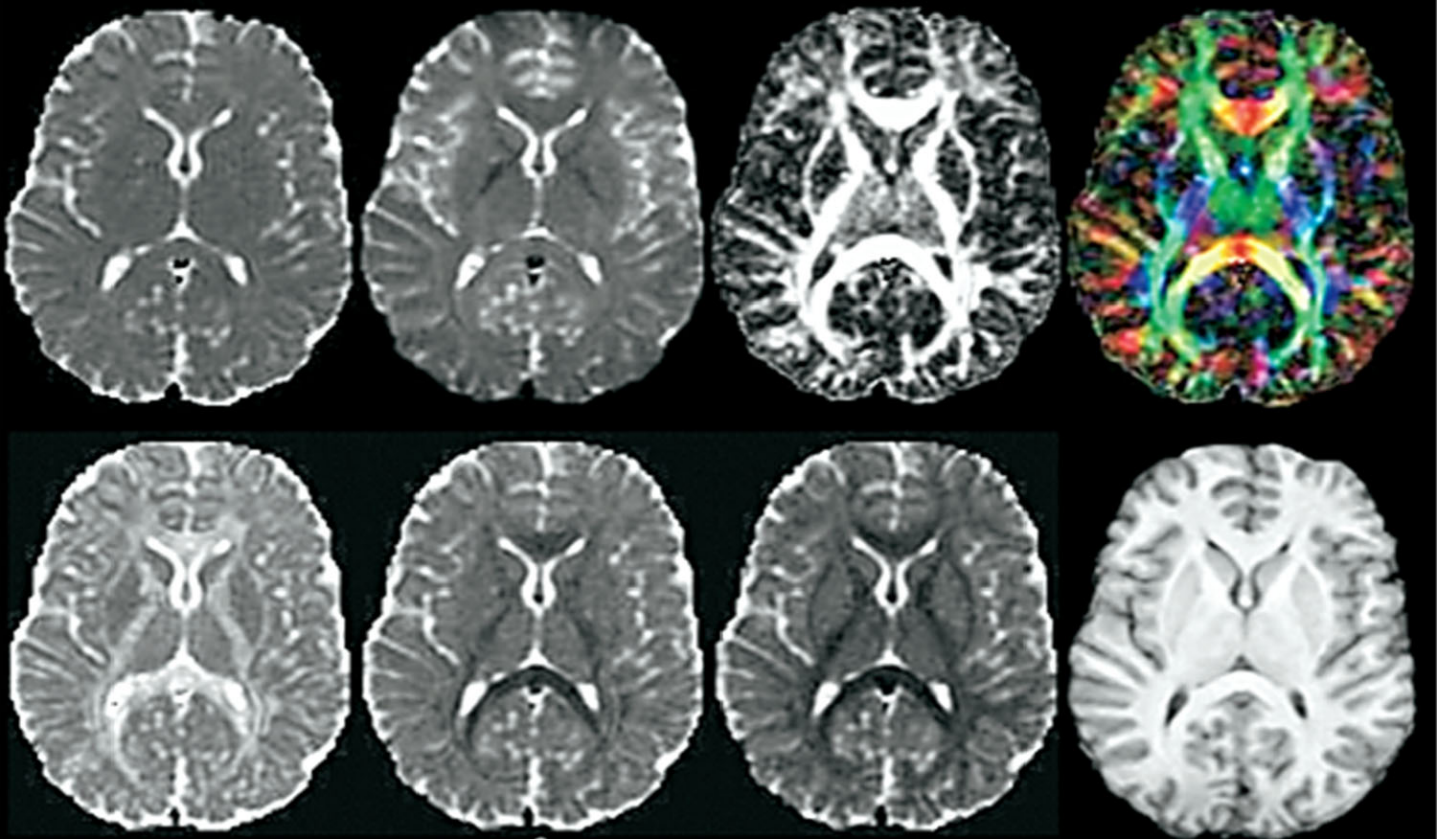
Different brain DTI images [22].

DTI monitors the movement of water molecules inside neural cells, facilitating the identification of white matter tracts and the evaluation of neuron connectivity stability. It aids in the examination of brain structure connections, the identification of anomalies in brain disorders, and the representation of cerebral systems [21]. Figure 5 shows the different brain DTI imaging.

### 3.4 SPECT Imaging

**Figure 6:**
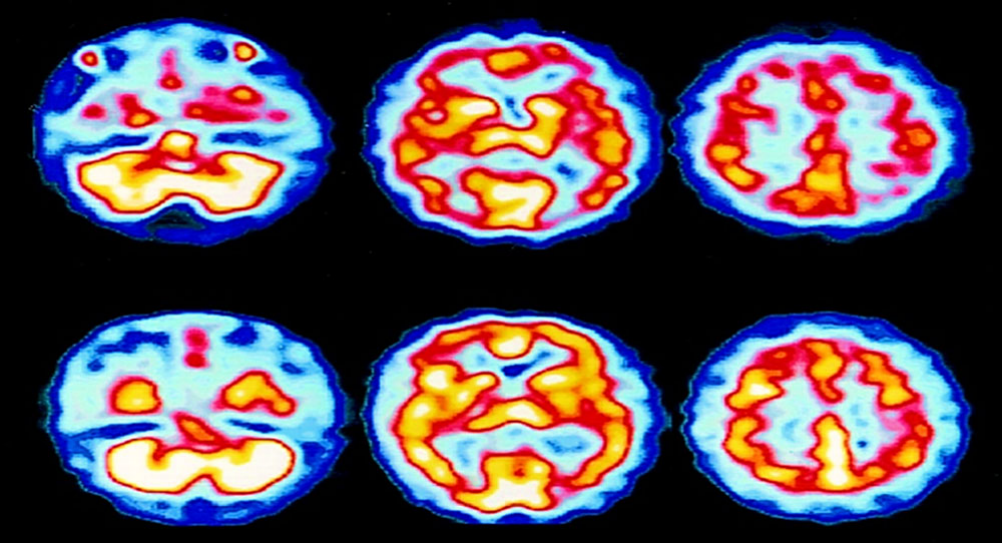
Brain SPECT images [24].

SPECT is more cost‐effective compared to alternative technologies, yet it is more sensitive while assessing variations in brain blood circulation for the very first time [23]. Despite this, in the analysis of brain activities, this technique remains one of the most often utilized approaches. Multiple tests indicate that SPECT can precisely evaluate brain perfusion in patients undergoing AD assessments [20]. Figure 6 shows the SPECT images.

### 3.5 PET Imaging

**Figure 7:**
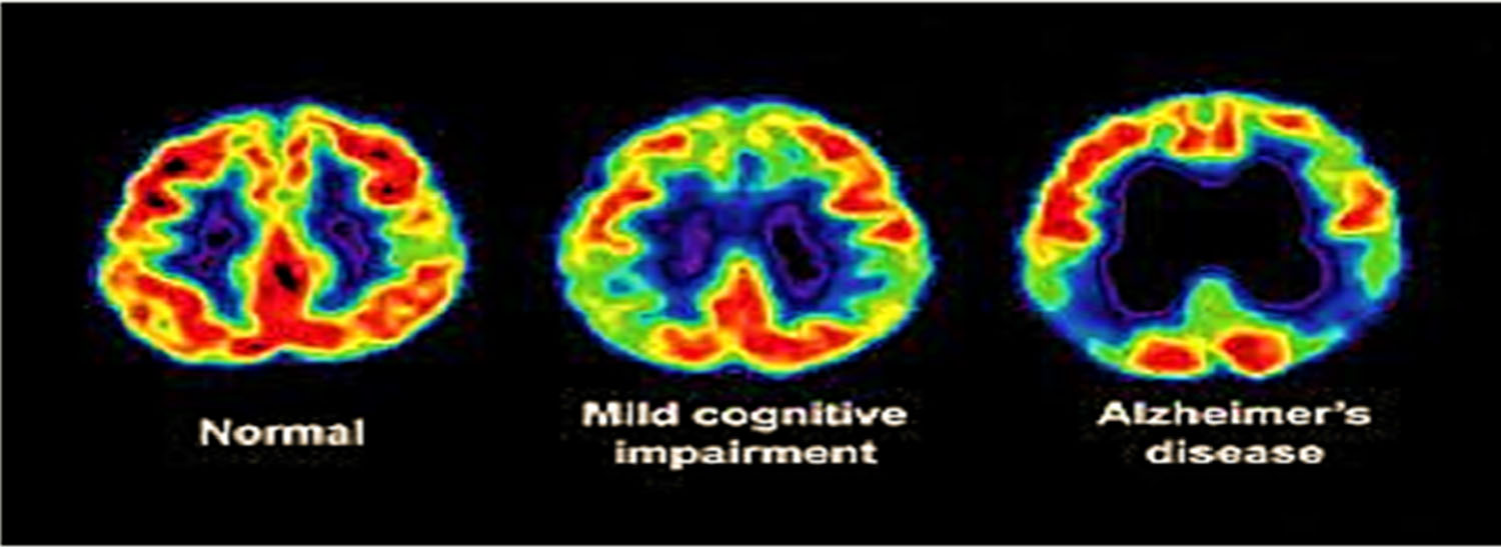
PET images of the brain left to right (normal, mild cognitive impairment, and AD) [11].

PET is an imaging method that assesses physiology by analyzing digestion, blood flow, radiolabelled drugs, and neurotransmitters [25]. PET images are crucial in diagnosing AD as they track the brain's glucose metabolism. PET imaging tests can detect biochemical anomalies, which helps doctors recognize the initial signs of AD and differentiate them from other forms of dementia [11]. Figure 7 shows the PET images of the brain.

### 3.6 Comparison of Imaging Modalities

MRI is essential for detecting AD because it offers excellent‐quality anatomical images that facilitate the evaluation of structural changes in the brain, especially in the area of the medial temporal lobes and hippocampus. DTI, a specialist MRI modality, improves diagnostic ability by delineating white matter stability and uncovering microstructural variations anticipating anticipated neurological signs. Although DTI and MRI are proficient in morphological evaluation, they do not provide an immediate understanding of the biochemical and functional abnormalities linked to the course of AD. These techniques are frequently employed alongside functional imaging to enhance the precision of diagnosis. Functional techniques for imaging, including PET, SPECT, and fMRI, offer essential physiological and biological information. PET, especially with amyloid, identifies pathologic particle accumulations in areas affected by AD. SPECT, while more economical, delivers less resolution compared to PET; however, it still yields significant neurological perfusion information. fMRI, conversely, assesses brain functioning through variations in oxygenation in the blood, facilitating the initial diagnosis of functional deterioration. Although fMRI as well as PET offer delicate functional information, their exorbitant costs and restricted availability prevent broad usage. A combined method that integrates functional and structural imaging provides the most effective screening procedure.

### 3.7 AD Detection Biomarkers

**Figure 8:**
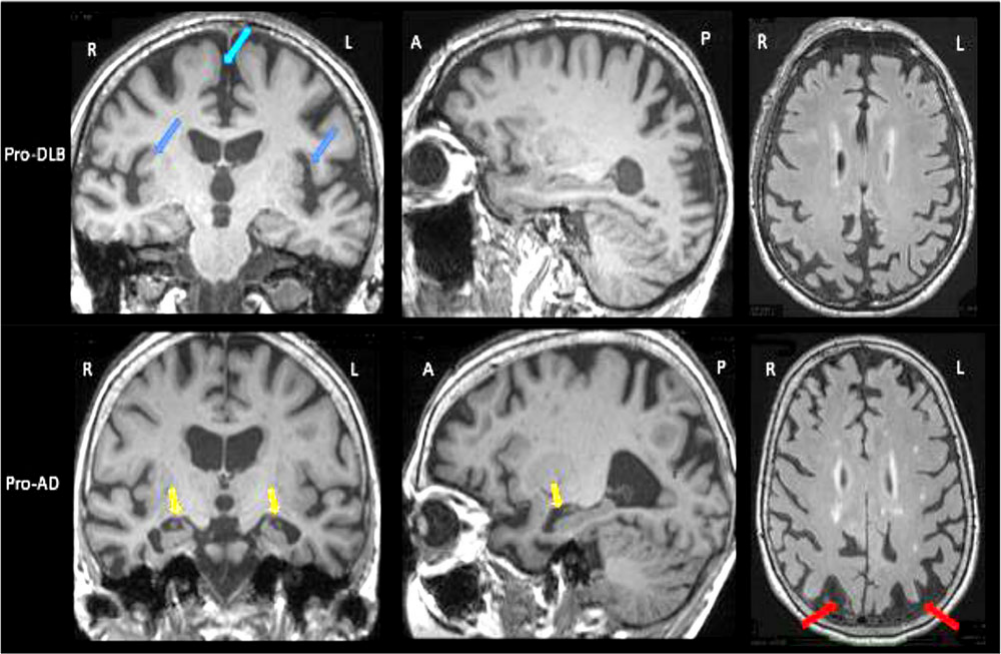
Biomarkers in MRI for AD.

Biomarkers are medical indicators that may be accurately assessed and are visible signs of an individual's bodily state. Biomarkers can be defined in multiple ways [26]. Hippocampal size, which has a tendency to diminish among individuals with AD, along with shrinkage of the middle temporal lobe, are significant MRI markers for AD [20]. Innovative MRI techniques may also be employed to visualize plaques of amyloid, which serve as an indicator of AD. The fMRI is a method used to analyze variations in brain function to detect regions that have been impacted by abnormalities connected to AD. DTI is a technique that evaluates the strength of WM in the brain and identifies any abnormalities linked to AD. Furthermore, fMRI assesses changes in connections within neural structures impacted by AD. The MRI biomarkers mentioned in the study allow for quick detection and tracking of AD development, helping in the timely discovery and understanding of its progression [27]. Figure 8 represents the biomarkers in MRI for AD.

## 4 Data Sets

A significant data set is necessary for identifying AD using DL. Data collection is a method used to gather information. A data set facilitates the examination of AD's advancement despite needing an actual patient. The primary databases accessible to academics studying AD are OASIS, AIBL, ADNI, Japanese ADNI, NACC, NRCD, and Kaggle, MICCAI, and IBSR data sets. The OASIS database was created through the AD research unit at the University of Washington and includes considerable neurological MRI data from individuals without illnesses and those with AD [28]. The ADNI data set contains data from MRI scans performed using 1.5 to 3 T scanner strength beams. As a result, this set of data is ideal for treating Alzheimer's illness [29]. The Neuromorphic Metrics Inc. of Scotts Valley in California employed 38 T1‐w MRI images and 135 physically segmented components to construct the MICCAI‐2012 database [30]. The IBSR data set evaluates and enhances the methods employed in segmenting brain images [31]. The acronym AIBL refers to the Australian Imaging Biomarker and Lifestyle landmark project in aging research. In the year 2006, for which AIBL was initialized [32]. Japanese ADNI is based on the ADNI database. ADNI was created in 2005, resulting in the same recruiting outcomes due to identical recruitment standards [33]. The Individuals who participate at the National Research Center for Dementia (NRCD) [34] in the Republic of South Korea undergo scanning utilizing sMRI. The NACC [35] comprises individuals who have organized interviews and data from neuroimaging. NACC was established in 2004, intending to use a joint data‐gathering strategy with 29 distinct AD centers. Kaggle [36] was established in 2010; Kaggle plate form has different Alzheimer's data sets with three, four, and five classes. It updated over time. The minimal interval resonance imaging in AD (MIRIAD) data set offers longitudinal T1 MRI to optimize AD research studies [37].

## 5 Alzheimer's Diagnosis With DL Approaches

DL is gaining significant interest in clinical studies due to its untapped potential for feature extraction, classification of data, and selection. DL is suggested for the initial AD detection due to its superior image accuracy for classification compared to the classic machine learning approach [38]. This article presented an AD diagnosis regarding supervised learning techniques and unsupervised learning techniques.

### 5.1 AD Diagnosis With Unsupervised DL

The use of training data for unsupervised learning has no labels. What is needed to be included in an extensive amount of unlabeled data? The objective of the training is to categorize or differentiate the information collected. When comparing a well‐organized feature extractor with professional expertise and manual architecture to a deep method. The following major unsupervised learning models are mostly used.

- Autoencoder (AE).
- Deep Belief Networks (DBNs).
- Generative Adversarial Networks (GANs).
- Restricted Boltzmann Machine (RBM).
- Variational Autoencoder (VAE).

Table 1 showed the different unsupervised models for AD detection.

**Table 1:** AD diagnosis research with unsupervised DL models.

References	Year	Data set	Image type	Method	Key findings
Marti‐Juan et al. [39]	2023	ADNI	MRI	Multi‐channel recurrent VAE	Relations among different modalities and model temporal data
Chen et al. [40]	2022	ADNI	Longitudinal DNA	Multi‐task deep Autoencoder	Rebuilding the chronological DNA methylation profiles
Jin et al. [41]	2021	ADNI	MRI	VAE and GAN	Can identify individual deteriorate in AD persons
Mansingh et al. [42]	2022	ADNI	MRI	Convolutional autoencoder	High accuracy
Kumar et al. [43]	2023	ADNI	MRI	Multi‐modal VAE	Abnormality maps are more delicate to disease staging
Li et al. [44]	2025	ADNI	MRI	Decoder adversarial autoencoder	Concentrate on definite disease areas
Sinha et al. [45]	2021	ADNI, AIBL	MRI	Attention‐guided GANs	Classification based on the non‐harmonized system
SinhaRoy et al. [46]	2024	ADNI	MRI	DCGANs and SRGANs	Producing synthetic MRI scans
Pan et al. [47]	2021	ADNI	fMRI, DTI	Hypergraph GAN	Detect discriminative brain areas of Alzheimer's
Lin et al. [48]	2021	ADNI	MRI, PET	Reversible GAN	Physical and functional information of brain tissue mapped fit
Valoor et al. [49]	2025	ADNI	MRI	U‐Net with GAN	Advantage in taking refined yet important structural features
Yu et al. [50]	2022	ADNI	MRI	Multidirectional perception GAN	Lesions visualized are consistent
Chadebec et al. [51]	2022	ADNI	MRI	Geometry‐based VAE	Applicable where small data size
Wang et al. [52]	2023	OASIS‐3	fMRI	Conditional VAE with adversarial learning	Show higher sensitivity to AD
Chai et al. [53]	2021	ADNI	MRI	Graph conditional VAE	Enhanced expected cortical thickness maps
Bandyopadhyay et al. [54]	2022	Clock data set	CDT	VAE	Increase the latent space of the VAE
Shi et al. [55]	2023	ADNI and Xuanwu cohort	MRI	Generative adversarial network constrained multiple loss AE	Enhance image reconstruction with the structural similarity index
Syed et al. [56]	2023	ADNI	MRI	Convolutional AE and deep parallel ensemble	To uncover important data patterns, and reveal patterns for AD
Safari [57]	2023	ADNI‐2	PET and MRI	Denoising AE stacked DL	Suitable to small sample learning issues and performs reliably
Menagadevi et al. [58]	2023	Kaggle, ADNI	MRI	Deep residual AE and SVM	Good classification accuracy
Jiao et al. [59]	2024	ADNI	fMRI, sMRI	Deep AE and self‐representation model	To assess genetic effects utilizing brain anatomy and function as phenotypes
Maity et al. [60]	2022	Kaggle	MRI	VAE model	Imbalance Alzheimer's detection
Bai et al. [61]	2022	ANDI, OASIS	MRI	Brain slice GAN	Extract high‐level brain features to improve diagnosis
Pei and Guan [62]	2021	ADNI	fMRI	RBM	Features extracted from fMRI increase accuracy by 7.5%
Chang et al. [63]	2022	ADNI	fMRI	RBM	The accuracy of the four classification tasks was 7.68%
John et al. [64]	2021	ADNI	PET	VAE	PET brain anomalies through assessing reconstruction error divergence
Minne et al. [65]	2022	ADNI	MRI	GAN‐based augmentation	Refining the diagnostic precision of numerous neurodegenerative sicknesses
Sekhar et al. [66]	2023	ADNI	PET, MRI	Ensemble models and GAN approach	Increase AD detection performance
Song et al. [67]	2021	ADNI	EEG	Generative adversarial learning	Physiological reasonability and the efficiency of produced data
Cabreza et al. [68]	2022	ADNI	sMRI	Unsupervised GAN learning	Anomaly recognition on brain MRIs to identify AD
Gao et al. [69]	2023	ADNI‐1, ADNI‐2, OASIS‐3	PET, MRI	Multi‐level guided GAN	Produce missing data using multiple levels of voxels and features
Hu et al. [70]	2023	ADNI	sMRI	VGG‐16 based CNN	Enhance detection accuracy over longitudinal sMRI
Miao et al. [71]	2024	ADNI	PET, MRI	Multi‐scale transformer fusion model	Transformer system that learns multi‐modal representations evenly
Li et al. [72]	2022	UK Biobank AIBL, ADNI	MRI	CNNs and transformers	Good for small data set
Lei et al. [73]	2023	ADNI	MRI	Federated domain adaptation structure via Transformer	Train networks using multi‐site data securely
Xing et al. [74]	2022	ADNI	PET	Vision Transformer (ViT)	Achieve good AUC value
Gao et al. [75]	2023	ADNI	sMRI	Multi‐scale attention convolution and aging transformer model	Capturing brain area local differences and long‐range associations
Yin et al. [76]	2022	ADNI	MRI	Multiple instance learning and self‐supervised ViT	Good classification accuracy compared to transformer
Pranav et al. [77]	2022	Pitt corpus data set	MRI	ViT method	Better differentiate between AD and normal
Sherwani et al. [78]	2023	ADNI	MRI	CNN and ViT	Training on fewer data sets shows inductive bias
Kadri et al. [79]	2021	ADNI and OASIS	MRI	Cross ViT and Wide Residual Squeeze‐Excitation model	Excellent accuracy and overcoming the limitation of data
Duan et al. [80]	2023	ADNI‐3	MRI	ViT through auxiliary branch	Solves the issue of dropping shallow features
Li et al. [81]	2023	ADNI	MRI	Mobile ViT	Effective and correct verdict in AD

#### 5.1.1 Limitations of Unsupervised Methods

Unsupervised learning techniques, however versatile and capable of detecting latent structures in data without specified labeling, possess some intrinsic shortcomings. These restrictions emerge from the complication of identifying significant representations in the absence of labeled assistance. After analyzing the above studies mentioned in the table, I have concluded that the AEs algorithms mostly provide ambiguous outputs, particularly in image‐related applications, and their latent space can remove critical data to reduce restoration loss [57, 60]. DBNs are highly computational and relatively inefficient compared to backpropagation from beginning to end. Inappropriate effectiveness on massive amounts of data relative to CNN models. These algorithms are mostly replaced with current methods [82, 83]. In GAN methodologies, the generator can provide a restricted range of data, and achieving stability between the generator and the discriminator behaviors needs rigorous hyper parameter optimization [68]. In RBM models, training by contrasting divergent yields inadequate gradient approximations, resulting in several iterations. Inappropriate for high‐dimensional information. The shallow design limits the acquisition of feature hierarchies [62, 63]. In VAEs, the Variational prior might not accurately show how the information is distributed and show poor separation without making more structural changes.

#### 5.1.2 Critical Analysis of Unsupervised Methods

The above Table 1 presents a diverse display of unsupervised DL methodologies, emphasizing the potential of AI to advance AD detection. AEs are exceptional at reducing dimensionality but cannot generate complicated data. The deterministic aspect of these frameworks restricts the adaptability of the sampling process, resulting in them being weaker to probabilistic methods in terms of generation. AE training is advantageous for implementations such as denoising; however, adversarial training has made it less effective. The importance of DBNs currently is confined to empirical research, while other options like GANs and VAEs provide stronger adaptability and training performance. Although producing excellent samples, GANs have challenges with repeatability and reliability. Exponentially developing solutions enhance outputs, yet their obscure nature restricts comprehensibility in essential tasks. RBM models established the foundation for energy based frameworks; however, they are impracticable for existing largescale applications. The combination of methodologies is present but does not possess the effectiveness of transformers or CNN. VAEs offer structured Bayesian systems that compromise the quality of samples for the sake of tractability. Hybrid designs integrate benefits despite gaining complications. Their precise probabilities enhance tasks like the detection of anomalies, whereas GANs struggle. Overall, every approach has unique compromises: VAEs and AEs emphasize consistency on sample quality, and GANs gain excellent accuracy but with increased training complexity, and RBMs and DBNs are mostly regarded as old benchmarks. The next steps will focus on hybrid systems that manage flexibility, stability, and interpretation.

### 5.2 AD Diagnosis With Supervised DL

In supervised DL, methods are learned to map input information into output labels using pairs of inputs and outputs from the training process. Supervised learning trains the mapping mechanism from input information to the intended outcomes, usually given by professional annotators. Supervised DL has excelled in natural language processing (NLP), computer vision, recognition of speech, and others. Supervised DL using 2D images involves DL methods to evaluate and retrieve data from 2D images within a supervised learning architecture. The details of different models used with 2D imaging in various domains are as follows.

- Convolutional Neural Network (CNN).
- Recurrent Neural Network (RNN).
- Gated Recurrent Unit (GRU).
- Capsule Networks (CapsNet).
- Long Short‐Term Memory (LSTM).
- Transformers.

Table 2 showed the different models used for supervised learning methods for AD detection.

**Table 2:** AD diagnosis research using supervised DL with 2D imaging.

References	Year	Data set	Image type	Method	Key Findings
Pandey et al. [84]	2025	Kaggle	MRI	ViT model	Improving MRI processing for AD analysis
Mehmood et al. [85]	2021	ADNI	MRI	Transfer learning	Excellent accuracy
Venkatasubramanian et al. [86]	2023	ADNI	MRI	DHO based pre‐trained CNN	Hippocampus segmentation and AD classification
Yan et al. [87]	2025	OASIS	MRI	ViT and ResNet‐50	Increase initial identification
Prasath and Sumathi [88]	2024	Kaggle	MRI	Adopted LeNet	A reduced execution length due to its design
Li et al. [89]	2025	Kaggle	MRI	Enhanced residual attention network	Classification accuracy of model enhanced
Abdelaziz et al. [90]	2021	ADNI	MRI, PET	CNN	Good in classification and regression tasks
Alsadhan [91]	2025	Kaggle	MRI	VGG16‐based model	Got better results in detection
Al‐Khuzaie et al. [92]	2021	OASIS	MRI	CNN	Excellent accuracy
Singh and Kumar [93]	2024	ADNI	MRI	Pre‐trained CNN models	Use significant memory concern class
Vasukidevi et al. [94]	2021	Kaggle	MRI	CapsNet	Average accuracy
Katkam et al. [95]	2025	MNDD	MRI	DenseNet‐169 & DeepLabV3+ model	Precise description of disease areas is efficiently segmented
Sindhu and Kumaratharan [96]	2025	ADNI	MRI	Taylor Dingo Optimizer model	Segmentation and classification
Janghel and Rathore [97]	2021	ADNI	fMRI and PET	Deep CNN	For feature extraction
Yao et al. [98]	2023	Kaggle & ROAD	MRI	Fuzzy‐VGG	Local image characteristics provide important information
Liu et al. [99]	2021	OASIS	MRI	Depth wise separable CNN	Employed for transfer learning less power consumption
Huang et al. [100]	2021	ADNI	PET	CNN + SVM Networks	Feature extraction classification
Ozdemir and Dogan [101]	2024	ADNI	MRI	Transfer learning techniques	Changed pooling layers with the Avg‐TopK technique
Ahila et al. [102]	2022	ADNI	PET	Deep learning neural networks	Better accuracy
Eroglu et al. [103]	2022	ADNI	MRI	Hybrid CNN	Features enhanced by the mRMR technique with good accuracy
Sener et al. [104]	2024	ADNI	MRI	DenseNet, EfficientNetB0, and AlexNet methods	Statistical McNamara's Experiment was used to fix the arithmetical importance
Sarkar [105]	2025	Real‐world data	MRI	Hybrid DL model	Ability to gather spatiotemporal dependences in data
Priyatama et al. [106]	2023	Kaggle	MRI	CNN and transfer learning	Four classes of AD
Amini et al. [107]	2022	ADNI	PET	Genetics and CNN	Better accuracy
Dhinagar et al. [108]	2023	OASIS3 & ADNI	MRI	CNN pre‐trained	Saliency records show the extra activation areas
Hagos et al. [109]	2025	ADNI	MRI & PET	Multi‐modal learners	Decreases the inference burden of the model
Al Shehri [110]	2022	Kaggle	MRI	Dense Net‐ 169 and ResNet‐50 CNN	DenseNet‐169 outperformed other approaches in training and testing
Khan et al. [111]	2025	Kaggle	MRI	YOLOv5 and YOLOv8	Improved sensitivity and accuracy compared to baseline architectures
Arafa et al. [112]	2024	Kaggle	MRI	VGG16 method	Suitable for basic organization with slight computing cost
Francis et al. [113]	2021	ADNI	MRI	CNN	Local feature descriptor applies rapid Hessian detector and local binary pattern for good work
Mahim et al. [114]	2024	ADNI	MRI	ViT and GRU model	Good for class inequity problem
Lanjewar et al. [115]	2023	Kaggle	MRI	CNN‐KNN framework	Performance has increased greatly over DCNN
Tanveer et al. [116]	2024	ADNI	MRI, PET	Ensemble deep learning	Initial interference and treatment arrangement and adds to increasing the knowledge
Ghadami and Rahebi [117]	2025	ADNI	MRI	CNNs and Harris Hawks Optimization	Showed better feature extraction
Arafa et al. [112]	2024	Kaggle	MRI	CNN and transfer learning	Suitable in low computing complexity, overfitting, storage use, and time control
Li et al. [118]	2021	ADNI	MRI	Cascaded CNNs	Hierarchical hippocampus forms and asymmetry from binary masking
Srividhya et al. [119]	2024	ADNI2	MRI	ResNet‐50v2	Better pre‐trained technique that can correctly diagnose
Kina [120]	2025		MRI	Efficient Net	Better in computing competence and generality
Zhou et al. [121]	2023	ADNI	MRI	CNN	Increase the performance of AD classification in fewer time
Hajamohideen et al. [122]	2023	ADNI, OASIS	MRI, PET, fMRI	Siamese CNN	Triplet‐loss function for the illustration MRI as k‐dimensional embedding's
Chabib et al. [123]	2023	Kaggle	CT & MRI	DeepCurvMRI	Capturing the vital structural variations in images
Kaur et al. [124]	2022	Kaggle	MRI	Deep CNN	False positive ratio and classification inaccuracy ratio are extremely small
Lin and Lin [125]	2021	OASIS	MRI	3D‐CNN	Uniform experimental design defines ideal systems parameters
Fathi et al. [126]	2024	ADNI	MRI	CNN‐based classifiers	Ensemble technique exhibited encouraging outputs
Mehmood et al. [127]	2022	ADNI	MRI	Two CNN based methods	Segmentation + methods provide advanced accurate outcomes for testing
Basheer et al. [128]	2021	OASIS	MRI, PET	Deep neural network	Suitable variables and feature selection
Balasundaram et al. [129]	2023	Kaggle & OASIS 2	MRI	Ensemble learning systems	Efficient in the initial identification of AD
Alahmad et al. [130]	2025	Kaggle	MRI	AlzONet	Minimize the loss function
Sethi et al. [131]	2021	ADNI	MRI	Deep convolution LSTM	Fewer iterations with a comparative enhancement
El‐Assy et al. [132]	2024	ADNI	MRI	CNNs	Taking appropriate attributes
Liang et al. [133]	2021	ADNI	MRI	Deep RNN	Good for missing value and prediction
Ji et al. [134]	2024	ADNI	fMRI	Convolutional bidirectional GRU	Superior classification performance
Kaplan et al. [135]	2023	Kaggle AD	MRI & CT	ExHiF method	Better accuracy
Shoeibi et al. [136]	2021	From institution	EEG Signals	CNN‐LSTM	Excellent accuracy achieved
Saravanakumar and Saravanan [137]	2022	Self‐collected	EEG	Stacked LSTM and CNN	Find temporal association among features
Aborokbah [138]	2024	OASIS & ADNI	MRI	EfficientNet‐B0 method	Decreases the complexity of the scheme and acquires a associated area
Asaduzzaman et al. [139]	2025	ADNI	MRI	Alzheimer Recognition Ensemble Network	Proficient tool for the initial and precise recognition of AD
Park et al. [140]	2022	ADNI	2D, 3D PET	CNN and LSTM	Multi‐modal classification
Zhao et al. [141]	2023	ADNI	MRI	16‐layer VGA and AlexNet techniques	Rise of accuracy 4% in several cases
Stefanou et al. [142]	2025	University Hospital of Thessaloniki	EEG	CNN	Effective, scalable on EEG
Dai et al. [143]	2022	ADNI, ABIDE, and MPILMBB	fMRI	CNN	Generalizability for several brain disorders
Rezaee et al. [144]	2025	Brazilian and Figshare	EEG	Cascade Net's	Handle overfitting well
Hassan et al. [145]	2025	Kaggle	MRI	Stacked CNN	Shows less parameters
Dhinagar et al. [146]	2023	UK Biobank (UKBB)	MRI	ViT	Analyzed the impact of the training data
Dhaygude et al. [147]	2024	ADNI	MRI	Advanced C3D system	Good binary accuracy
Chen et al. [148]	2025	ADNI	Medical record, MRI	ViT	Resolve the class imbalance problem
Sen et al. [149]	2023	ADNI	MRI	ViT	Shows good performance
Hassan et al. [150]	2025	ADNI	MRI	Gradual Variation Based Dual Stream DL model	Robust and computational efficient
Sarraf et al. [151]	2023	ADNI	sMRI	Optimized ViT	It has 30% less parameters
Kadri et al. [152]	2022	ADNI, OASIS	MRI, PET	EfficientNet2 and ViT	Strong feature mining and representation
Vanaja et al. [153]	2025	Kaggle and ADNI	MRI	Customized DCNN	Different cohorts and features from images
Khan et al. [154]	2024	ADNI	MRI, PET	Mixed Transformer	Multi head attention mechanism to decrease feature size
Pan and Wang [155]	2022	ADNI	DTI, fMRI	Cross‐modal transformer GAN	Balancing information among structural and functional features
Prasad et al. [156]	2025	ADNI	MRI	Directed acyclic graph 3D‐CNN	Regions of interest helps in AD detection
Zia‐Ur‐Rehman et al. [157]	2024	ADNI	MRI	DenseNet‐201	Good accuracy on five classes of AD
Tuncer et al. [158]	2025	Kaggle	MRI	CNN FiboNeX	Present next generation CNN
Shin et al. [159].	2023	Dong‐A University cohort	PET	ViT	Probably owing to under‐induction and overfitting bias
Mahjoubi et al. [160]	2025	ADNI	MRI	ResNet50	Emphasizes on loss, accuracy, and time
Odusami et al. [161]	2023	ADNI	MRI, PET	Pixel‐Level Fusion and ViT	The precision of detecting AD can increase with integrated data
Rafsan et al. [162]	2025	OASIS	MRI	Bayesian CNN and U‐net	Good for imbalance data
Zheng et al. [163]	2022	ADNI	MRI	Transformer‐based multi‐features fusion	Prediction of MCI transformation with 83.3% accuracy
Miltiadous et al. [164]	2023	General University Hospital of Thessaloniki	EEG	Dual input convolution encoder model	Efficiently capture the multifaceted features
Zhang et al. [165]	2025	China Aging and Neuro Degenerative Initiative	MRI, PET, MMSE	Fully CNN	Categorize randomly image patches
Lyu et al. [166]	2022	ADNI	MRI	ViT	Efficiently transmission the knowledge learned in the normal image
Abdelaziz et al. [167]	2025	ADNI	MRI, PET	Multimodal and multi‐scale model	Better performance compared to other models
Drewitt [168]	2023	Kaggle	MRI	ViT	Used in four classes
Al‐Adhaileh [169]	2022	Kaggle	MRI	Alex Net 19 and Restnet50	The suggested technique can help increase CAD Approaches for AD in clinical investigations
Basher et al. [170]	2021	Gwangju Alzheimer's and Related Dementia	sMRI	Discrete volume estimation CNN	Extracted volumetric data of each slice linked to the hippocampus of sMRI
Balakrishnan et al. [171]	2025	ADNI	MRI	RNN	Achieved good accuracy
Kushol et al. [172]	2022	ADNI	MRI	Fusion Transformer	Good classification accuracy
Bhatele et al. [173]	2022	ADPP, and ADNI	MRI	VGG19, VGG16ResNet50 InceptionV3	Achieves enhanced as related to former prevalent deep transfer learning methods
Neha Shivhare et al. [174]	2022	Dementia databank	Audio files	LSTM, GRU	Information on Alzheimer's prediction in a single language can be shifted to a different language
Beatrice and Dhivviyanandnam [175]	2025	ADNI	MRI, PET, DTI, and fMRI,	CNNs model	Make a merged demonstration that preserves the important features
Suresha and Parthasarathy [176]	2021	OASIS, NIH, and Neuro Sciences databases.	MRI	PCA and LSTM	Showed a 3%–11% enhancement in recognition correctness
Alinsaif et al. [177]	2025	ADNI	MRI	Four DL models	Association exploration for enriched feature
Alessandrini et al. [178]	2022	Hospitalized data	EEG	Robust‐PCA and LSTM RNN	RPCA was capable of enhancing the recognition accuracy by 5%
Ye et al. [179]	2025	ADNI and OASIS	MRI	Multi scale spatial self attention model	Reduce scale parameters
Tang et al. [180]	2024	ADNI	sMRI and PET	3D‐CNN	Increases the interpretability of the method
Goel et al. [181]	2025	OASIS	MRI	Self‐adaptive evolutionary	Distinguish deteriorates and iron accumulation
Gharaibeh et al. [182]	2023	ADNI	MRI	Segmentation using Modified U‐Net, GAN	Features attention in terms of channel and spatial that enhance the detection accuracy
Nisha et al. [183]	2025	ADNI 2	MRI	Attention BiLSTM	Feature mining, segmentation, and feature choice

#### 5.2.1 Limitations of Supervised Methods

The Table 2 includes research that demonstrates a diverse display of supervised DL methodologies utilized for the diagnosis of AD by MRI, PET, and several different types of imaging. Nevertheless, several techniques encounter difficulties. CNN methods have difficulties with spatial changes because of their static size of filters and pools of layers that remove spatial hierarchy. Although translational invariance assists in identifying objects, it restricts accurate localization tasks. These models need extensive data sets with labels and significant computer resources; however, slight changes can deceive these techniques [121]. Analysis of RNN models reveals their difficulty in managing persistent dependencies that are intrinsically non‐parallelizable, resulting in protracted training times and reduced storage space for lengthy sequencing [170, 171]. GRU algorithms have less parameters compared to LSTM models, perhaps restricting their ability to record complicated patterns; at present, these techniques continue to face challenges with exceedingly lengthy sequences [114]. CapsNet methods require significant amounts of resources. This paradigm possesses advantages and disadvantages, including inferior empirical outcomes on extensive data sets. Conversely, real‐life scenarios require empirical confirmation of the advantages of these methods, such as perspective invariance [94, 184]. Because they have a lot of hidden states, LSTM methods use a lot of memory, and the fact that they cannot be parallelized slows down processing. Susceptible to remembering noise in limited data sets [136, 137]. The Transformers technique employs attention that grows with sequential size, whereas global attention can ignore local features. These methodologies need large data sets for the pretaining task [172]. Although CNNs and RNNs, different networks, established the foundation, the flexibility, reliability, and effectiveness restrictions drove attention toward the Transformer mechanism and mixed models. GRU and CapsNet techniques have potential; however, they need empirical verification. The discussion emphasizes the necessity for systems that balance theoretical precision, computing speed, and practical application.

#### 5.2.2 Critical Analysis of Supervised Methods

CNN techniques are proficient with grid structured input that needs a theoretical foundation for managing geometrical variances. Although structural advancements persist in being data‐intensive and unstable. Solutions for this, such as CapsNet technique aim to fix these challenges but have not yet achieved broad adoption. RNN methodologies were once popular in modeling sequences and have recently been dominated by the Transformers, among others. Its sequencing properties and gradient instabilities restrict the ability to scale. Hybrid systems provide limited solutions but do not achieve the performance of transformers. GRU architectures compromise intricacy for speed; despite this, the benefits are minimal for today's massive applications. Their acceptance decreases as Transformers prevail, while they continue to be beneficial in situations with limited resources. The claim of geometric stability in CapsNet models is still unverified. The absence of viable alternatives and issues with repeatability limit the advancement of these techniques. LSTM methods were crucial in model sequencing and have become specialized because of the emergence of transformers as well. Its gate strategies enhance flow with gradients but introduce intricacy without assurances of improved generality. Transformers have transformed NLP but encounter issues related to scaling and interpretation. Combined approaches and sparse attention mitigate these challenges; however, theoretical deficiencies remain in explaining attention strategy.

### 5.3 DL With 3D Imaging

The details of different DL models used with 3D imaging are presented here. Previous studies indicated that there was limited work on 3D imaging with DL approaches; however, some researchers employed the 3D characteristics of models that were not exclusively focused on 3D imaging but rather leveraged these abilities. Medical experts employ MRI scans to build 3‐D images of the body's interior architecture. Therefore, in many studies, its importance increased when 3D was used in AD prediction and diagnosis, as Zhang et al. [185] designed a dense neural network system extracted multiple scale features from processed data, and a connection‐wise attention process combined features from various levels to create dense advanced maps of features. Wu et al. [186] introduced a ResNet3D‐18‐based 3D CNN framework for multiclass classification, which investigated learning‐based AD progression detection.

Khagi and Kwon [187] presented research that found a slight improvement in the cerebral spinal fluid in brain ventricles and sulci, a significant reduction in GM content, and decreased brain size in AD individuals. Castellano et al. [188] proposed a one and multi‐modal architecture and method that focuses on important AD related areas for its estimations. Rasheed et al. [189] introduced a Caps Net, which takes fewer data for training while providing improved results for classification. The study introduced a new computer assisted method for the early stages of Alzheimer's detection using an explainable 3D residual attention deep neural system for learning directly from sMRI data [190]. Akan et al. [191] presented a ViTranZheimer, an AD diagnostic method that analyzes 3D brain MRI scans using video vision transformers. In evaluating measures, ViTranZheimer was the most reliable. Dhinagar et al. [192] presented a 3D CNN framework that can identify neurological disorders. In addition, disparities in Parkinson's disease and AD classification assignment learning and test efficiency were found for the suggested framework. Gao et al. [193] proposed research that introduces the 3DMgNet design, a multigrid and CNN structure for AD diagnosis. AD versus NC classification accuracy was only 2%, and model parameters were significantly dropped.

Zia‐Ur‐Rehman et al. [194] proposed first ever research which solely provide insights regarding 3D imaging with DL methods which showed that limited work on three planes images which give accurate predictions for AD. Narazani et al. [195] provided an approach for evaluating multi‐modal DNNs, comparing single and multipurpose networks employing FDG‐PET and sMRI for binary and three‐way classifications. Zuo et al. [196] compared visual attention among AD individuals and normal and used a nonintrusive eye‐tracking method to capture heat maps of visual attention throughout a 3D extensive visual assignment. Swarun Raj et al. [197] utilizes a multi layered 3D CNN structure to effectively build ordered representations from the temporal and spatial attributes of data. Zhang et al. [198] introduced a ResNet‐based end‐to‐end 3D CNN model for AD detection, using multi‐layer characteristics gained via the attention process to detect tiny variations in brain. Researchers introduced an experimental process for MRI‐based categorization of AD versus CN individuals, prioritizing reliability and consistency [199]. Researchers presented a feature evaluation and categorization are done using a DCNN and 3D‐DCNN [200].

Tuan et al. [201] proposed a novel computational technique for diagnosing AD using a 3D MRI of the brain. The effective method involves classification and segmentation, each using DL. Atalar et al. [202] suggested a graphic‐based DL technique facilitates initial AD diagnosis. The data is fused by merging textual data with 3D MRI using visual pipelines. Tufail et al. [203] investigated the influence of three data augmentation strategies on the effectiveness of CNN frameworks in 3D for the initial detection of AD. Rao et al. [204] presented a transfer learning on 3D MRI for multi‐class classification of AD and achieved excellent outcomes. Researchers delivered a complete approach using 3D Convolutional LSTM for the initial detection of Alzheimer's using full‐resolution whole‐brain structural MRI [205]. In Ayyar et al. [206], the technique adapts a newly published feature‐based clarification to classify AD and NC in 3D CNN structures using hippocampal regions of interest from brain structural MRIs. This study introduced a 3D CNN for identifying AD using amyloid PET images, a less‐explored study area, and an investigation using the newly published OASIS‐3 data [207].

Kim et al. [208] research proposed a middle‐fusion multi‐modal approach for the initial detection of AD, and the method gathers features without any loss. The research uses a 3D CNN to diagnose AD by identifying intricate patterns in MRI data [209]. Ismail et al. [210] presented a multimodal image fusion method that automatically fuses and converts ADNI neuroimaging data into the BIDS standard for classifying Alzheimer's patients from controls. Hogan and Christoforou [211] aim to determine whether 3D CNNs can distinguish neurophysiological deterioration in entire‐brain imaging among AD patients and unaffected individuals. Chen et al. [212] proposed a slice‐level attentiveness technique highlighting selected 2D slices and eliminating repetition. Next, a 3D CNN was used to document global subject‐level changes in structure. Balboni et al. used a transfer‐learning model on a previously trained spatially warping segmentation of networks. People with moderate MCI and AD were analyzed using MRI scans segmented using two separate ratings [213].

### 5.4 Limitations and Critical Analysis of DL With 3D Imaging

The extensive, excellent 3D imaging data sets are limited because of substantial acquisition expenses, legal and ethical issues. There is also necessity for long‐term studies. The class discrepancy could bias techniques in favor of the most prevalent categories. Labeling for the initial stages of AD is particularly difficult because of their nuanced or confusing image characteristics. Resource‐constrained environments cannot access 3D image data due to its processing and memory requirements. Imaging similarities across AD and different dementia types can misclassify them [214]. 3D scans include confidential patient data, demanding rigorous anonymity and safe storing measures. Although DL with 3D images shows potential for AD detection, overcoming these challenges needs cooperative initiatives to enhance the quality of data, system transparency, efficiency in computation, and healthcare synergy. Additionally, integrated methodologies, collaborative learning, and strict external verification are essential for practical application [11]. DL has markedly improved AD diagnosis with 3D imaging techniques, including PET and MRI, utilizing CNNs and transformer‐based techniques for automatically obtaining features and categorization. These approaches demonstrate better capability in detecting complex anatomical and operational abnormalities in the brain linked to AD, surpassing conventional techniques in both precision and effectiveness. Still, limitations remain, such as the necessity for extensive, meticulously annotated data sets, substantial computing costs, and the opaque nature of DL algorithms, constraining interpretability in clinical environments. Moreover, discrepancies in imaging methods and demographic factors may affect the generalizability of models. Addressing these challenges necessitates using explainable AI methodologies, domain adaptation methods, and hybrid fusion tactics to improve the stability and clinical relevance of DL in Alzheimer's identification.

## 6 Recent Advances From 2021 to 2025 Compared to Earlier Research

From the years 2021 to 2025, there have been significant developments in AD research in comparison to previous decades. During this period, there was a notable advancement in identifying advanced imaging methods like PET and MRI, which have facilitated earlier and more accurate detection. Researchers are also looking into new biomarkers, such as indicators found in blood and cerebrospinal fluid, which could help find AD early on. Moreover, most research focuses on MRI and PET imaging modalities because of their superior ability to detect AD. However, researchers are increasingly exploring other imaging modalities such as DTI and EEG, which can significantly contribute to AD research. After analyzing various techniques for AD diagnosis, we found that most research using CNN and its various variants has both pros and cons. On the other hand, researchers are now mostly focusing on ViTs and their diverse types, which show significant performance in the imaging domain and in AD detection. By taking only important parts and features from the images with a reduced number of different parameters and providing good performance, although it needs good hardware and software for the processing of those images, we can solve these problems with a good design of models and features‐obtaining procedures.

## 7 Discussion

The use of DL algorithms has recently seen a considerable increase in the diagnosis and prediction of AD, demonstrating promising outcomes in many areas. Identifying the progression between MCI and Alzheimer's and recognizing the early phase of individuals having MCI are advantageous for the diagnosis and evaluation of Alzheimer's. Experts have consistently discovered three main subcategories by rigorous neuropathological and radiological examinations: normal AD, hippocampal‐sparing AD, and limbic‐predominant AD, as well as the development of the fourth subcategory, mild shrinkage AD. Experts have identified a subcategory of Alzheimer's illness that lacks observable shrinkage. The various subcategories have been identified by intricate structures of shrinkage of the brain and neuropathological features, displaying diverse medical and psychological traits, including some types showing reduced development of the disease relative to the normal AD appearance [215]. In terms of performance, DL surpasses conventional machine learning techniques. DL enhances healthcare efficiency and decreases healthcare costs, which remains under discussion. The main problem is the deficiency of studies on the analysis of images related to AD pathology. Improving the DL methodology represents a paramount breakthrough in enhancing these networks [216]. The effectiveness of the Alzheimer's illness detection method is significantly reliant on the precision of brain imaging. Patch‐based and area‐of‐concern techniques are essential. The integration of biological data, cognitive assessment evaluations, cerebrospinal fluid indicators, and characteristics derived from brain neural imaging algorithms can facilitate precise categorization. The evaluation of this article reveals that DL has been extensively utilized in Alzheimer's disorder identification. Clinical imaging research indicates that full training of CNN models has become the preferred approach in recent decades [217]. DL methods, namely CNNs and RNNs, have been used to analyze data from multiple sources, including neuroimaging images, genetic information, and medical records. These methods have demonstrated significant achievements in identifying disorders at initial stages, categorizing different disease phases, and predicting disease development. The research effort in this domain will persist regardless of an extension in the data set because its effectiveness is still ambiguous. Furthermore, neuroimaging annotations are another challenge. Radiology professionals must annotate the photographic data offered for particular purposes when labeling clinical images. The image classification procedure is exceedingly time‐intensive. A supervised learning strategy successfully addressed this issue, reducing the need for professional knowledge; however, more study remains crucial [218, 219, 220, 221, 222]. The 2D imaging is simple, affordable, and widely available. Its fast view capture is ideal for healthcare assessments, industrial checks, and scientific research. High‐quality 2D images allow a comprehensive representation of objects or structures. In medical diagnosis and object identification, depth of information is vital but limited. 3D imaging improves visualization and perception of depth, facilitating healthcare diagnosis, architectural design, and simulations. Its comprehensive spatial data allows better assessments and evaluations than 2D imaging. However, data analysis and understanding are more complicated, software and hardware could cost more, and accessibility and interoperability with present systems are issues [223, 224, 225, 226, 227]. Brain imaging research showed that using algorithms for classification and neuroimaging techniques could help identify AD during the early stages before clinical signs and symptoms become apparent. Consequently, multiple study organizations are dedicated to categorizing AD and MCI by applying different neuroimaging techniques, including MRI, fMRI, and PET. The application of DL for categorizing AD has significantly improved.

## 8 Challenges

Although DL techniques have shown positive outcomes, several challenges still exist that need to be handled in identifying AD. Despite the fact that the data augmentation and transfer learning models have prevented overfitting problems in the research and group of information, the absence of adequate data items can continue to result in issues with generalization. To overcome this challenge, data generation approaches could produce fresh data from current ones. The data set will be expanded; however, its efficiency remains uncertain, and more studies will continue in this domain. CNN models face challenges when generalizing various types of imaging and acquisition strategies. Alzheimer's research generally includes multi‐site cooperation and inconsistencies in neuroimaging regulations, which could result in undesirable variability. Establishing effective methodologies to address these problems and assure the generalization of models is a critical research domain. GANs provide potential for data enhancement, synthetic imaging, and creating accurate images of the brain. Addressing difficulties in this area is also necessary. Accessibility to huge and varied data sets for training GANs for investigations into AD might be challenging with respect to concerns regarding privacy and data accessibility. It is crucial to produce photographs that are biologically plausible in nature and accurately depict the fundamental pathology of AD. The current study aims to balance augmenting data with protecting the authenticity of AD‐specific properties. Image annotation presents an additional barrier. Radiologists must modify healthcare images by labeling the available information for specific purposes. Supervised DL has mitigated this issue and decreased the reliance on professional expertise. Despite the impressive achievements of DL methods, the area of AD identification currently needs several restrictions and challenges. A comprehensive understanding of the depth method, benchmarking platform, and other relevant factors is necessary to determine the ideal balance of several biomarkers. The extent to which it may decrease healthcare costs and enhance medical efficiency has yet to be thoroughly validated. It is important to address the challenges associated with limited data availability in the AD investigation. DL techniques often require large amounts of labeled data for optimal performance. Still, Alzheimer's data are often limited, with obstacles and costs related to acquiring data. Several fusing approaches have the potential to contribute to research on AD, which is the focus of our upcoming study. Still, there are obstacles to overcome when using DL‐based techniques to tackle AD. These issues include dealing with different data types, ensuring the results can be easily understood, and applying the methods to varied populations. Furthermore, Current initiatives have concentrated on advancing explainable AI methodologies, including saliency maps and attention strategies, to elucidate the predictive processes of systems.

## 9 Conclusion

The early identification of AD remains a challenge, and computer scientists are continuously investigating potential solutions. This study mainly presents the different imaging modalities, data sets used for AD diagnosis, supervised and unsupervised DL, 2D and 3D imaging applications to identify AD, and their comprehensive analysis. DL approaches improve AD diagnosis and prediction, allowing for early detection and personalized treatment. DL techniques have shown success in using multiple data modalities. In contrast with various other DL methods, CNN models are the most frequently employed and important classification method in this field. The issue of overfitting in data endures and requires a solution. The study areas of healthcare images change due to self‐management and unsupervised processes resulting from insufficient clinical data. Despite several unresolved issues regarding the classification of AD, the DL system remains significantly effective. Transfer learning methods and integrative methodologies have significantly improved predictive powers, minimizing reliance on extensive data sets with labels. Moreover, the optimal results were demonstrated by the integration of the basic CNN model with MR brain images. MRI is the most common technique employed for the classification of AD. Using complementary data from different imaging types provides better AD detection performance than relying on only one imaging technique. In comparison to voxel and slice‐based techniques, strategies for processing region‐of‐interest and patch‐based data have proven much more effective. DL approaches represent the cutting edge of innovation and are increasingly transforming the global strategy for AD diagnostics. Presently, fMRI DL techniques exhibit great accuracy; however, they remain developing and need much more investigation. This study's results highlight the capability of DL to enhance AD detection precision. It is crucial to understand that DL is not a single remedy; it must be combined with different medical data and detection methods to get thorough and efficient AD identification.

## 10 Future Directions

The future studies will need to focus on enhancing ways to increase the accessibility and explication of DL algorithms used in the early prediction and detection of AD. Even though most issues in the area of AD classification remain unsolved, the interpretation in healthcare fields is crucial for gaining physician confidence and adoption, and it ensures the ethical deployment of AI technological advances. Establishing standardized norms and assessment parameters for DL initiatives related to AD is recommended to enhance cooperation and accelerate progress in the area. Furthermore, the distribution of carefully selected and annotation‐rich data sets reduces the difficulties associated with limited data and fosters the development of new techniques and methodologies. To resolve this problem, investigators can explore transfer learning approaches for the investigation of AD. Additionally, the issue of overfitting pertaining to the data set needs solutions. The use of data augmentation methodologies could synthetically increase the number and range of available data, hence helping in the generation of more secure and applicable systems. Moreover, CNN algorithms have difficulties extrapolating over various types of imaging and collecting techniques. AD research often includes multi‐site cooperation and discrepancies in imaging techniques, which may cause undesirable variability. Novel GAN models can also be vital in generation and image processing tasks, especially healthcare imaging. The professional and experienced radiologist provides a more authentic data set, which can ultimately offer better performance in different metrics. Formulating effective methodologies to address these issues and ensure the generalization of the model is a critical research focus. Security constraints and data accessibility make it difficult to collect significant and varied data sets for AD studies using GAN models. Making sure that the images are physiologically accurate and represent AD disease is also problematic. Current studies seek to match augmenting data with AD‐related attribute reliability. More cost‐effective and improved fusion techniques can present enhanced results in various domains. Designing federated learning methods could solve privacy issues and facilitate cooperative study on extensive databases. Furthermore, the exploration of explainable AI methodologies is essential to enhance the interpretability of models and foster confidence across doctors. Managing inefficiencies in data sets using synthesized data creation and area adaption methods can improve system resilience. Moreover, incorporating continuous surveillance tools employing mobile devices and cognitive evaluations with brain imaging information may provide an improved and individualized strategy for predicting AD. Integrating these developments with initiatives to guarantee ethical artificial intelligence implementation will influence the future of DL in neuroimaging‐based AD diagnosis. The initiatives listed above have the potential to change medical care and promote the establishment of individualized and specific treatments for people at risk or suffering from Alzheimer's illness.

## Author Contributions

**Zia‐ur‐Rehman:** conceptualization, methodology, writing – original draft, data curation, writing – review and editing, software. **Mohd Khalid Awang:** investigation, validation, formal analysis, supervision, project administration. **Ghulam Ali:** resources, writing – review and editing, supervision, validation, investigation. **Muhammad Faheem:** writing – review and editing, formal analysis, data curation, validation.

## Ethics Statement

This study did not include any actual human or animal subjects.

## Conflicts of Interest

The authors declare no conflicts of interest.

## Transparency Statement

The authors, affirm that this manuscript is an honest, accurate, and transparent account of the study being reported; that no important aspects of the study have been omitted; and that any discrepancies from the study as planned (and, if relevant, registered) have been explained.

## Data Availability

Data will be provided on reasonable request.
